# Occlusion Outcomes in Unilateral Amblyopia Types: A Longitudinal and Interventional Study in Children from the Marrakech-Safi Region

**DOI:** 10.22599/bioj.382

**Published:** 2024-12-27

**Authors:** Mustapha Jaouhari, Chaimae El Harrak, Farida Bentayeb, Youssef El Merabet

**Affiliations:** 1 Laboratory of Electronic Systems, Mechanical, and Energy Information Processing, MA; 2 Laboratory of high energy physics, modeling, and simulation, MA

**Keywords:** Amblyopia, Anisometropia, Strabismus, Occlusion, Visual recovery, Child, Eye patch, Visual acuity

## Abstract

**Objective:**

To investigate if the type of unilateral amblyopia can impact the improvement of visual acuity in amblyopic eyes during a longitudinal interventional study involving standard occlusion therapy in children.

**Methods:**

A longitudinal and interventional study of 91 children, aged 3–9 years (6.12 ± 1.879) with amblyopia was undertaken. Amblyopia was unilateral and caused by either strabismus, anisometropia, or both. Amblyopia was divided into three levels of severity mild, moderate, and severe. Children underwent amblyopia treatment with standard occlusion therapy and were followed monthly, for eight visits.

**Results:**

A significant improvement in visual acuity was observed in all assessment visits across the three types of amblyopia, with a mean improvement of (0.30 ± 0.184) LogMAR. Specifically, anisometropic amblyopia with (0.28 ± 0.18) LogMAR, strabismic amblyopia (0.31 ± 0.18) LogMAR, and mixed amblyopia (0.31 ± 0.18) LogMAR. No significant differences were found between the improvement in visual acuity during the eight control visits and the types of amblyopia (r = 0.174, p = 0.182). However, a significant correlation was observed within all groups in the mean improvement in visual acuity and the severity level (r = 0.712, p = 0.034).

**Conclusion:**

Amblyopia types do not appear to significantly affect the improvement in visual acuity; however, the initial severity of amblyopia may be a key factor influencing the degree of visual recovery achieved with occlusion therapy.

## Introduction

Amblyopia is a reduction in corrected visual acuity (VA) in an eye with no apparent organic cause, (Holmes and Clarke, 2006). It is a common neurodevelopmental abnormality that results in physiological alterations of the visual pathways in one eye, less often in both (Levi, 2020). Amblyopia is common in childhood, with a global prevalence between 1.27% and 1.46% (Hu et al., 2022). It is more common in school-age children (DeSantis, 2014), primarily developing during the first 7–8 years of a child’s life during the visual pathway maturation period, known as the ‘critical period.’ In certain situations, the critical period may extend beyond eight years (Hensch and Quinlan, 2018), and any abnormalities during this time can impede the brain’s ability to appropriately assimilate visual signals from each eye.

Amblyopia is typically categorised as strabismic, in the presence of manifest strabismus (Zahir et al., 2023), or anisometropic when there is a difference in refractive error between the eyes (greater than or equal to 1DS or 2DC) (Chen et al., 2022). Mixed amblyopia results from two or more amblyogenic factors. Anisometropic and strabismic amblyopia is frequently observed, especially in cases of partially accommodative esotropia, microtropia, and monofixation syndrome (Lyu et al., 2021).

The treatment of amblyopia is typically ‘patching therapy’ or ‘occlusion therapy’ (Stewart et al., 2007), or atropine occlusion. Occlusion therapy has been the standard treatment for more than a century (Moseley et al., 2023) and consists of obstructing the better seeing eye to enable the amblyopic eye to view the world and receive ‘visual stimulation.’ Recent studies have explored novel techniques and approaches in occlusion therapy for amblyopia, focussing on the dose-response relationship and often evaluating their effectiveness based on factors such as patient age and amblyopia severity. However, this study aimed to compare the outcomes of occlusion therapy for different types of amblyopia (strabismic, anisometropic, and mixed types).

## Methods

The study obtained approval from the Biomedical Research Ethics Committee of Oujda (CERBO) and adhered to the principles of the Helsinki Declaration. The Moroccan Ministry of Health and Social Protection granted approval for this study under number 2324/2024. Prior to participation, all participants received thorough explanations about the study’s objectives, the importance of their involvement, and their right to decline participation. Assent was obtained from the children, and written informed consent was obtained from the legal guardian of each child prior to participation. All collected data were anonymised to ensure the removal of any personally identifiable information. The study took place in the Marrakech-Safi region, in a provincial hospital where patients from urban and rural areas are seen.

This was a longitudinal intervention study involving children, aged 3–9 years, who received occlusion therapy for unilateral amblyopia of various types and varying levels of severity. Unilateral amblyopia was defined as best corrected VA worse than 0.2 LogMAR in the amblyopic eye, with 0.2 LogMAR difference or more between the two eyes.

Inclusion criteria were the presence of strabismic, anisometropic or mixed amblyopia. Additionally, participants were required to have knowledge of the extent of glasses worn prior to the trial, as well as sufficient cognitive, motor, and verbal maturity to undergo visual acuity testing using letter optotypes. Exclusion criteria included any eye disease (including deprivation amblyopia), previous occlusion treatment for amblyopia, and developmental delay.

Each type of amblyopia within the three main groups (anisometropic, strabismic, and mixed) was further divided into three severity levels, as classified by a dosage scheme based on a study conducted by senior orthoptists, identified by members of the British and Irish Orthoptic Society. In this study, amblyopia was categorised as mild (better than 0.4 LogMAR), moderate (0.4 to less than 0.8 LogMAR), or severe (equal to or worse than 0.8 LogMAR) (Moseley et al., 2023).

### Examination and refractive adaptation

Prior to diagnosis of unilateral amblyopia all participants underwent an ophthalmological examination, orthoptic examination and refraction under full cycloplegia. Participants with refractive error were prescribed full correction for astigmatism, anisometropic myopia, and mild to moderate hypermetropia (+2.00 to +5.00), and within two diopters of the full correction for high hypermetropic errors.

Instances when refractive correction was not prescribed included bilateral hypermetropia or myopia of 1.50 DS or less, and bilateral astigmatism of 0.75 DC or less.

If refractive correction (glasses) was prescribed, all participants wore glasses for 18 weeks prior to starting occlusion therapy (Stewart et al, 2004; Hong et al, 2024). Glasses wear was full time. Children were seen at six-week intervals for reassessment during refractive adaptation. Children showing improvements between weeks 12 and 18 continued refractive adaptation for six-week intervals, until no further gains in visual acuity in the amblyopic eye were measured. The duration of this refractive adaptation phase was 18 weeks: a period chosen based on the studies of (Stewart, 2004; Hong et al., 2024) indicating that no clinically significant gain (0.10 LogMAR) occurred beyond this period. If no refractive correction was required, occlusion therapy was started immediately.

### Occlusion therapy

A standard model of occlusion therapy was used for all participants (Moseley et al., 2023). One of three dosage schemes was assigned based on VA at the end of refractive adaptation (if required). The prescribed dosages were mild amblyopia (<0.4 LogMAR): two hours per day, moderate amblyopia (0.4 to <0.8 LogMAR): three hours per day, and severe amblyopia (≥0.8 LogMAR): five hours per day.

Visual acuity was measured after refractive adaptation (if required) and prior to starting occlusion therapy was referred to as the baseline VA. Following this, VA was measured monthly, and the rhythm of occlusion therapy was adjusted based on the patient’s VA if required. This approach was standardised for all patients.

### Statistical analysis

Data analyses were conducted using SPSS (version 23.0, IBM USA), with the significance level set at a P value < 0.05. The chi-square test was employed to compare categorical variables among the three amblyopia groups, specifically for sex distribution and living environment. One-way ANOVA was used to analyse the mean age across the three types of amblyopia (anisometropic, strabismic, and mixed). A two-way ANOVA was utilised to compare VA improvement between the three types of amblyopia and across the three levels of severity (mild, moderate, and severe). Bonferroni correction was applied for post-hoc comparisons to ensure statistical significance between intra-group differences.

## Results

The study included 91 children, mean age (6.12 ± 1.879 years) with a gender disparity of 67.03% boys and 32.96% girls (Table 2), from two different environments: 63% urban and 37% rural representing a unilateral amblyopia of three types: anisometropic (36.2%), strabismic (24.1%), and mixed (39.5%). The sample size was determined based on practical considerations, as no formal sample size calculation was performed. Tables 1, 2, and 3 present the characteristics of each child in its amblyopia type group.

**Table 1:** Characteristics of the study patients: gender, age, and place of origin.


SEX		AGE						

		3	4	5	6	7	8	9

**Female**	**rural**	1	4	4	2	2	3	2

	**urban**	1	1	1	3	2	3	1

	**Total**	2	5	5	5	4	6	3

**Male**	**rural**	0	2	2	3	3	4	2

	**urban**	6	9	5	5	7	9	4

	**Total**	6	11	7	8	10	13	6

**Total**	**rural**	1	6	6	5	5	7	4

	**urban**	7	10	6	8	9	12	5

	**Total**	8	16	12	13	14	19	9


**Table 2:** Demographic and baseline characteristics of the three amblyopia groups.


CHARACTERISTIC	STRABISMIC AMBLYOPIA GROUP (n= 22)	ANISOMETROPIC AMBLYOPIA GROUP (n= 36)	MIXED AMBLYOPIA GROUP (n= 33)

**Age Range**	3 to 9 years	3 to 9 years	3 to 9 years

**Mean Age**	5.454 ± 2.109	6.25 ± 1.74642492	6.303 ± 1.9761

**Gender Distribution**	7 female, 15 male	12 female, 24 male	11 female, 22 male

**Environment**	16 Urban, 6 Rural	20 Urban, 16 Rural	23 Urban, 10 Rural

**Baseline Mean VA (Amblyopic Eye) LogMAR**	0.60 ± 0.27	0.63 ± 0.25640	0.577 ± 0.25640

**Baseline Mean VA (Better Eye) LogMAR**	0.029 ± 0.033	0.0251 ± 0.034	0.028 ± 0.04

**Baseline Mean Refractive Error (Amblyopic Eye)**	Mean sphere: –1.79 ± 3.14	Mean sphere: –4.92 ± 5.26	Mean sphere: –4.46 ± 4.18

	Range sphere: –5.25 to +3.25	Range sphere: –9 to +6	Range sphere: –9 to +6

	Mean cylinder: –0.95 ± 0.82	Mean cylinder: –1.33 ±0.98	Mean cylinder: –0.97 ± 0.85

	Range cylinder: 0 to –2	Range cylinder: 0 to –3.50	Range cylinder: 0 to –4

**Baseline Mean Refractive Error (Better Eye)**	Mean sphere: –1.6 ± 2.805	Mean sphere: –0.93 ± 2.26	Mean sphere: –1.11 ± 1.92

	Range sphere: –5.25 to +3	Range sphere: –6 to +5	Range sphere: –3 to +4

	Mean cylinder: –1.16 ± 1.04	Mean cylinder: –0.52 ± 0.66	Mean cylinder: –0.88 ± 0.89

	Range cylinder: 0 to –2.75	Range cylinder: 0 to –2.75	Range cylinder: 0 to –2.75

**Severity**	Moderate: 14	Moderate: 20	Moderate: 20

	Mild: 4	Mild: 7	Mild: 8

	Severe:4	Severe:9	Severe:5


**Table 3:** Comparison of best-corrected distance VA of amblyopic eye between the three groups of amblyopia and the three level of severity. Values are mean ± SD.


ASSESSMENT MONTH	SEVERITY LEVEL	STRABISMIC AMBLYOPIA GROUP (n = 22) – MEAN VA (LogMAR)	ANISOMETROPIC AMBLYOPIA GROUP (n = 36) – MEAN VA (LogMAR)	MIXED AMBLYOPIA GROUP (n = 33) – MEAN VA (LogMAR)	P VALUE BETWEEN GROUPS

**Baseline**	**Total**	0.60 ± 0.27	0.63 ± 0.25640	0.577 ± 0.25640	<0.001¹

	**Mild**	0.20 ± 0.062	0.212 ± 0.06	0.25 ± 0.05	<0.001¹

	**Moderate**	0.63 ± 0.097	0.664 ± 0.09	0.61 ± 0.10	<0.003¹

	**Severe**	0.96 ± 0.054	0.975 ± 0.05	0.96 ± 0.05	<0.003¹

	**P value** **Intra-groups**	0.25^2^	0.361^2^	0.910^2^	

**Month 1**	**Mild**	0.13 ± 0.051	0.12 ± 0.05	0.17 ± 0.04	<0.001¹

	**Moderate**	0.52 ± 0.052	0.542 ± 0.08	0.52 ± 0.06	<0.003¹

	**severe**	0.88 ± 0.044	0.875 ± 0.05	0.87 ± 0.06	<0.003¹

	**P value** **Intra-groups**	0.288^2^	0.599^2^	0.992^2^	

**Month 2**	**Mild**	0.08 ± 0,035	0.1 ± 0.01	0.10 ± 0.01	<0.001¹

	**Moderate**	0.42 ± 0,052	0.414 ± 0.05	0.4 ± 0.01	<0.001¹

	**Severe**	0.76 ± 0,054	0.75 ± 0.05	0.74 ± 0.07	<0.003¹

	**P value** **Intra-groups**	0.53^2^	0.310^2^	0.914^2^	

**Month 3**	**Mild**	0.03 ± 0.051	0.025 ± 0.005	0.05 ± 0.05	<0.001¹

	**Moderate**	0.36 ± 0.058	0.378 ± 0.04	0.37 ± 0.04	<0.003¹

	**Severe**	0.76 ± 0.140	0.575 ± 0.15	0.57 ± 0.13	<0.001¹

	**P value** **Intra-groups**	0.593^2^	0.745^2^	0.266^2^	

**Month 4**	**Mild**	0	0		–

	**Moderate**	0.30 ± 0.051	0.292 ± 0.02	0.3 ± 0.032	<0.001¹

	**Severe**	0.62 ± 0.100	0.55 ± 0.1	0.54 ± 0.08	<0.001¹

	**P value** **Intra-groups**	0.288^2^	0.677^2^	0.562^2^	

**Month 5**	**Mild**	0	0		–

	**Moderate**	0.26 ± 0.067	0.278 ± 0.05	0.25 ± 0.06	<0.001¹

	**Severe**	0.52 ± 0.083	0.45 ± 0.1	0.42 ± 0.08	<0.001¹

	**P value** **Intra-groups**	0.488^2^	0.599^2^	0.165^2^	

**Month 6**	**Mild**	0	0		–

	**Moderate**	0.16 ± 0.048	0.178 ± 0.04	0.17 ± 0.44	<0.001¹

	**Severe**	0.29 ± 0.048	0.28 ± 0.04	0.27 ± 0.44	<0.003¹

	**P value** **Intra-groups**	0.068^2^	0.658^2^	0.658^2^	

**Month 7**	**Mild**	0	0		–

	**Moderate**	0.01 ± 0.030	_	0.005 ± 0.2	<0.001¹

	**Severe**	0.28 ± 0.100	0.4 ± 0.05	0.31 ± 0.10	<0.003¹

	**P value** **Intra-groups**	0.18^2^	0.467^2^	0.188^2^	

**Month 8**	**Mild**	0	0		–

	**Moderate**	0.005 ± 0.02	_	_	<0.001¹

	**Severe**	0.24 ± 0.054	0.37 ± 0.05	0.30 ± 0.10	<0.003¹

	**P value** **Intra-groups**	0.878^2^	0.435^2^	0.077^2^	


Note: ^1^One way anova test between groups (to compare VA improvement for the same severity level across different types of amblyopia), ^2^Two-way anova test intra-groups. (to compare VA improvement across different severity levels (mild, moderate, severe) within the same amblyopia type.

There was a significant improvement in VA across the three amblyopia groups from baseline acuity after the refractive period, to the acuity measured in assessment at visit eight, with a mean improvement of 0.30 ± 0.18 LogMAR. Specifically, in the anisometropic amblyopia group a mean improvement of 0.28 ± 0.18 LogMAR, the strabismic amblyopia group with 0.31 ± 0.18 LogMAR, and the mixed group 0.31 ± 0.18 LogMAR.

**Figure 1:**
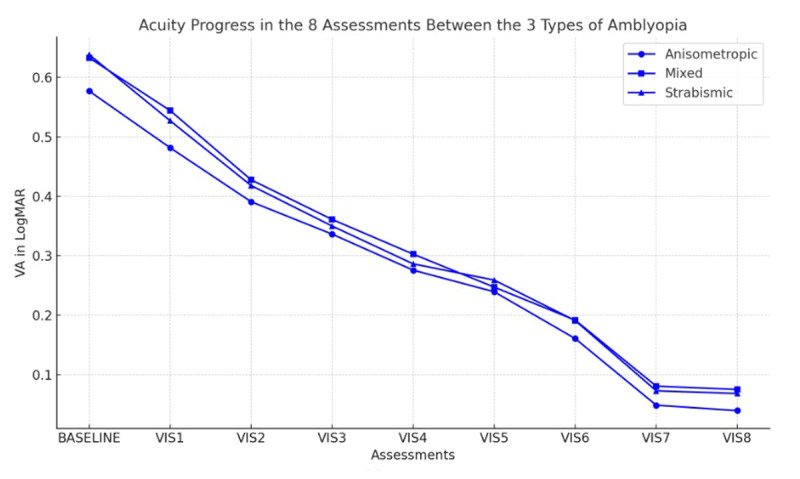
Measurements of VA in the three types of amblyopia across the eight assessments.

**Figure 2:**
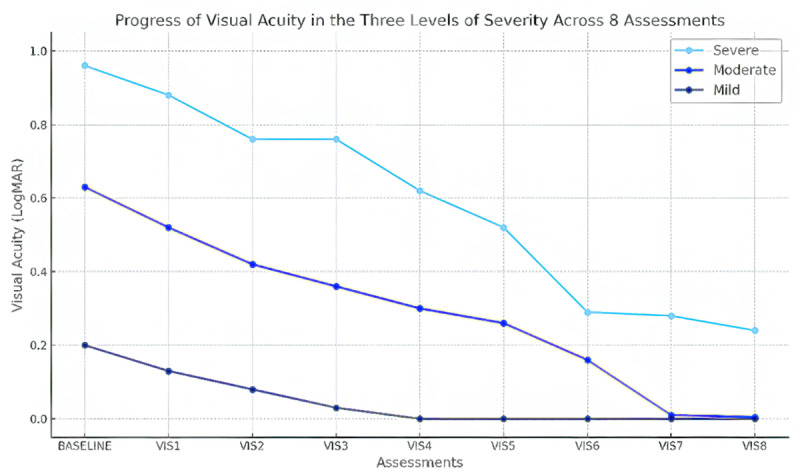
Measurements of VA in the three severity levels of amblyopia across the eight assessments.

Improvement in VA was also observed across all severity levels for each type of amblyopia, severe amblyopia showed a gain of 0.58 ± 0.08 LogMAR, moderate amblyopia improved by 0.29 ± 0.05 LogMAR, mild amblyopia demonstrated a smaller increase of 0.072 ± 0.05 LogMAR (Table 3, Figures 1, 2).

The statistical analysis found no significant differences in age between the amblyopia groups (F = 12, p = 0.679), and no significant differences in gender distribution across the groups (X^2^ = 0.017, df = 2, p = 0.991). Similarly, there were no significant differences between the children’s original environments (urban or rural) and the three types of amblyopia (X^2^ = 1.289, df = 2, p = 0.528).

No significant correlation was found between the improvement in VA over eight visits and the type of amblyopia (r = 0.174, p = 0.182). However, there was a significant correlation between the amount of VA improvement and the severity of amblyopia (r = 0.712, p = 0.034) (Table 3).

## Discussion

The results obtained in this study (shown in Table 3 and Figure 1) indicate that there was no significant difference in improvement of VA in different types of amblyopia. However, a significant difference was observed in the improvement in VA among the three severity levels within each amblyopia group (p = 0.034) as shown in Figure 2. There was a greater amount of improvement in VA in the amblyopic eye in those with more severe amblyopia. This may reflect the starting VA in the amblyopic eye, or it may reflect the different treatment protocols used in different severity of amblyopia, or both.

While we compared our findings with those from other studies, we acknowledge that differences in study design, treatment duration, and the categorisation of amblyopia types may influence the results. Our study followed a standardised treatment protocol with a consistent follow-up period of eight visits, which may differ from other studies that varied in treatment duration and intensity. Although few studies examined the relationship between amblyopia types and improved acuity (Mostafaie et al., 2020). Our results are similar to two previous Paediatric Eye Disease Investigator Group studies (2002; 2008) that have shown no significant relationship between the type of amblyopia and the improvement of VA. Stewart et al. (2004; 2007) have also previously found no difference in mean improvement in VA between types of strabismic, anisometropic and mixed amblyopia. Our results are in contrast to those from a South Korean study (Shim et al., 2017) investigating the factors that influenced amblyopia treatment outcomes. In 48 children from 2–13 years of age, amblyopia type did not affect the VA outcome or duration of treatment, but in children older than six years of age, those with higher refractive error and those with worse starting VA required longer durations of amblyopia treatment.

Those with worse starting VA, or more severe amblyopia, had greater improvements in VA, which was encouraging. This has been reported by others (Arikan et al., 2005; Stewart et al., 2005). The mild and moderate amblyopia groups in our study achieved end VA results that were not significantly different (Table 3, Figure 2), however the severe amblyopia group had a residual level of amblyopia in comparison. It is not clear from these results whether further occlusion would lead to further VA improvements.

A strength of our methods was the standardisation of an amblyopia treatment protocol following a similar approach to others conducting amblyopia research (PEDIG, 2002; PEDIG, 2008). However, we acknowledge there are limitations to this study. Patients with a spherical refractive difference of 1.00 DS between the two eyes were not included in the anisometropic amblyopia category. We used greater than 1.00 DS difference, which has led to lower levels of anisometropia not being included in the study. We did not measure other parameters, such as stereoacuity or ocular motor skills, which limits our ability to compare our results to other sources of evidence investigating these outcomes following amblyopia therapy.

## Conclusion

The treatment of unilateral amblyopia in children aged 3–9 years, with a standardised occlusion regime, led to similar improvements in VA in anisometropic, strabismic and mixed amblyopia. Those with more severe amblyopia at the start of treatment had greater improvements in VA.
